# The prognostic significance of allelic imbalance at key chromosomal loci in oral cancer

**DOI:** 10.1038/sj.bjc.6990290

**Published:** 1999-04

**Authors:** M Partridge, G Emilion, S Pateromichelakis, R A'Hern, G Lee, E Phillips, J Langdon

**Affiliations:** Maxillofacial Unit, Kings College School of Medicine and Dentistry, Denmark Hill, London, SE5 8RX, UK; Royal Marsden NHS Trust, Fulham Road, London, SW3 6JJ, UK

**Keywords:** oral cancer, chromosome deletions, genes, suppressor, loss of heterozygosity

## Abstract

Forty-eight primary oral squamous cell carcinomas (SCC) were screened for allelic imbalance (AI) at 3p24–26, 3p21, 3p13, 8p21–23, 9p21, 9q22 and within the Rb, p53 and DCC tumour suppressor genes. AI was detected at all TNM stages with stage 4 tumours showing significantly more aberrations than stage 1–3. A factional allelic loss (FAL) score was calculated for all tumours and a high score was associated with development of local recurrence (*P* = 0.033) and reduced survival (*P* = 0.0006). AI at one or more loci within the 3p24–26, 3p21, 3p13 and 9p21 regions or within the THRB and DCC genes was associated with reduced survival. The hazard ratios for survival analysis revealed that patients with AI at 3p24–26, 3p13 and 9p21 have an approximately 25 times increase in their mortality rate relative to a patient retaining heterozygosity at these loci. AI at specific pairs of loci, D3S686 and D9S171 and involving at least two of D3S1296, DCC and D9S43, was a better predictor of prognosis than the FAL score or TNM stage. These data suggest that it will be possible to develop a molecular staging system which will be a better predict of outcome than conventional clinicopathological features as the molecular events represent fundamental biological characteristics of each tumour. © 1999 Cancer Research Campaign


*British Journal of Cancer* (1999) **79**(11/12), 1821–1827
© 1999 Cancer Research Campaign
Article no. bjoc.1998.0290

### The prognostic significance of allelic imbalance at key chromosomal loci in oral cancer

**M Partridge[1], G Emilion[1], S Pateromichelakis[1], R A'Hern[2], G Lee[1], E Phillips[1] and J Langdon[1]**

[1]Maxillofacial Unit, Kings College School of Medicine and Dentistry, Denmark Hill, London SE5 8RX, UK; [2]Royal Marsden NHS Trust, Fulham Road, London SW3 6JJ, UK

**Summary** Forty-eight primary oral squamous cell carcinomas (SCC) were screened for allelic imbalance (AI) at 3p24–26, 3p21, 3p13, 8p21–23, 9p21, 9q22 and within the Rb, p53 and DCC tumour suppressor genes. AI was detected at all TNM stages with stage 4 tumours showing significantly more aberrations than stage 1–3. A factional allelic loss (FAL) score was calculated for all tumours and a high score was associated with development of local recurrence ($P = 0.033$) and reduced survival ($P = 0.0006$). AI at one or more loci within the 3p24–26, 3p21, 3p13 and 9p21 regions or within the THRB and DCC genes was associated with reduced survival. The hazard ratios for survival analysis revealed that patients with AI at 3p24–26, 3p13 and 9p21 have an approximately 25 times increase in their mortality rate relative to a patient retaining heterozygosity at these loci. AI at specific pairs of loci, D3S686 and D9S171 and involving at least two of D3S1296, DCC and D9S43, was a better predictor of prognosis than the FAL score or TNM stage. These data suggest that it will be possible to develop a molecular staging system which will be a better predict of outcome than conventional clinicopathological features as the molecular events represent fundamental biological characteristics of each tumour.

**Keywords:** oral cancer; chromosome deletions; genes; suppressor; loss of heterozygosity

The TNM tumour classification system together with tumour site, type, grade and thickness, are all used to predict which patients are at risk of developing local or distant recurrence and plan adjuvant therapy (Spiro et al, 1986; Anneroth et al, 1987; Langdon and Henk 1995). However, despite taking into account all the available prognostic information when making crucial decisions about treatment, many patients develop local or distant recurrence. This failure is due to the inability to detect small numbers of malignant cells which remain in the body after treatment and the requirement to take into account information about tumour biology in addition to the conventional clinical and histologic prognostic criteria.

Solid tumour formation requires multiple mutational events. Recent studies examining oral squamous cell carcinomas (SCC) have identified frequent deletion or mutation affecting one allele of the p53, Rb and DCC tumour suppressor genes (Largey et al, 1993; Lee et al 1994; Min et al, 1994; Brennan et al, 1995; Rowley et al, 1995; Maestro et al, 1996; Nylander et al, 1996). However, few studies have interrogated both copies of these sequences although homozygous deletions of p16 have been reported (Cairns et al, 1995). Several other chromosomal areas that are likely to harbour other suppressor sequences likely to play a role in the development of these tumours have been identified at chromosomes 3p, 8p and 9p by cytogenetics and loss of heterozygosity studies (El-Naggar et al, 1993; Field et al, 1994; Kiaris et al, 1994; Partridge et al, 1994, 1996; van der Reit et al, 1994; van Dyke et al, 1994; Wu et al, 1994; Califarno et al, 1996; Ishwad et al, 1996); however, the critical sequences within these candidate suppressor areas are currently unknown. Although only preliminary data about the genetic aberrations associated with tumorigenesis is available, a genetic model has been proposed (Califarno et al, 1996) with specific gene alterations indicated as early (AI at 3p, 9p21), intermediate (AI at 11q, 14q, 14q) and late events (AI at 8p, 4q). Study of head and neck (Field et al, 1994; El-Naggar et al, 1995; Partridge et al, 1996; Lee et al, 1997; Kelker et al, 1996; Lydiatt et al, 1998) and of other solid tumours (Kerangueven et al, 1997) has suggested that AI at specific chromosomal regions, and the number of aberrations detected, can help predict outcome (Vogelstein et al, 1989; Field et al, 1995). This new knowledge means that it is now possible to apply molecular studies to tumours in vitro and incorporate information about the key genetic abnormalities for each tumour into conventional staging systems. This will enable clinicians to identify a tumour's potential for progression more accurately and use this information to modify existing treatment protocols to improve clinical course and outcome.

In this study we have looked for allelic imbalance (AI) at 19 loci at chromosomes 3p, 8p21–23, 9p13–21, 9q22, 13q14.2 and within the p53, Rb and DCC tumour suppressor genes since these sequences have all been implicated in the pathogenesis of head and neck and oral SCC (see references cited above). We have used more markers at 3p since alterations here are the most frequent genetic change in oral cancer reported to date and the available evidence suggests that this chromosome arm may harbour multiple tumour suppressors (Field et al, 1994; Partridge et al, 1994, 1996; Wu et al, 1994; Ishwad et al, 1996). We have determined the prevalence of AI at these key loci and summarized the data as a fractional allelic loss (FAL; Vogelstein et al, 1989) score for each individual patient and looked for commonly recurring patterns of

*Received 1 May 1998*
*Revised 4 September 1998*
*Accepted 11 September 1998*

*Correspondence to:* M Partridge

**Table 1** Clinicopathological features and risk factors for oral SCC examined and alleleo-typing results of tumours examined

| Case | Sex | Age | Site | TNM | Stage | Histopathology | Smoking (cigarettes per day) | Alcohol (units per day) | Alive/dead | Cause of death | Survival time (m) | Recurrence (Y/N) | Disease-free survival (months) | D3S192 | THRB | D3S686 | D3S32 | D3S1241 | D3S1296 | D3S1562 | D3S30 | D8S261 | D8S298 | Ank 1 | D9S162 | INF alpha | D9S171 | D9S43 | D9S177 | Rb1 | TP53 | DCC | Number of loci AI | Number loci informative | FAL score |
|---|---|---|---|---|---|---|---|---|---|---|---|---|---|---|---|---|---|---|---|---|---|---|---|---|---|---|---|---|---|---|---|---|---|---|---|
| 1 | M | 78 | Alveolus | | | V | 40 | 12 | D | DC | 15 | N | 15 | ▨ | | ▨ | ▨ | ■ | ■ | ▨ | ■ | ▨ | ▨ | | ▨ | ■ | | ■ | ▨ | ■ | ▨ | ■ | 7 | 10 | 0.7 |
| 2 | M | 76 | Alveolus | | | V | 5 | 4 | D | DD | 48 | N | 48 | ▨ | | | ■ | ▨ | ▨ | • | ▨ | ▨ | ▨ | ▨ | ▨ | ▨ | ▨ | ▨ | ▨ | ▨ | ▨ | ▨ | 1 | 3 | 0.3 |
| 3 | M | 48 | Palate | | | V | 30 | 0 | A | | 48 | N | 48 | ▨ | • | ▨ | | | • | • | | • | | • | | • | | • | • | | | ▨ | 0 | 8 | 0 |
| 4 | F | 80 | FOM | | | V | 0 | 0 | D | DD | 24 | N | 24 | | • | | | ■ | ▨ | ■ | ▨ | ▨ | | ▨ | | | | | | | | | 2 | 14 | 0.14 |
| 5 | F | 79 | Tongue | T1N0 | 1 | W | 0 | 0 | A | | 120 | Y | 23 | ▨ | | | | ▨ | ■ | • | | ▨ | | | | | ▨ | ■ | ▨ | | ▨ | ▨ | 2 | 11 | 0.18 |
| 6 | F | 59 | Tongue | T1N0 | 1 | W | 0 | 0 | D | DD | 63 | Y | 36 | | | | ▨ | | | • | ▨ | ■ | ■ | ▨ | ■ | ■ | | ■ | | | ■ | ▨ | 6 | 14 | 0.42 |
| 7 | F | 48 | Buccal | T1N0 | 1 | W | 0 | 0 | A | | 60 | Y | 60 | ▨ | | ▨ | ▨ | | ▨ | ▨ | ■ | ▨ | ▨ | | | ■ | | | | | | ■ | 3 | 12 | 0.25 |
| 8 | M | 70 | Buccal | T1N0 | 1 | W | 0 | 0 | D | DD | 57 | Y | 26 | ■ | | | | | | ■ | | ▨ | ▨ | ▨ | ■ | | ▨ | | ▨ | | ■ | | 4 | 14 | 0.28 |
| 9 | M | 40 | FOM | T1N0 | 1 | W | 3 | 8 | D | DD | 24 | Y | 7 | | | ■ | | ▨ | | ■ | ▨ | | | ▨ | | | ■ | ■ | | | | ■ | 5 | 16 | 0.31 |
| 10 | M | 88 | Buccal | T1N0 | 1 | M | 20 | 0 | A | | 36 | N | 36 | | | | | | • | • | ▨ | ▨ | ■ | • | ▨ | ■ | | ■ | | | ▨ | ▨ | 3 | 11 | 0.27 |
| 11 | F | 70 | Tongue | T1N0 | 1 | M | 0 | 0 | A | | 36 | N | 36 | | • | | | | | ▨ | | ▨ | ■ | | | | | | | ▨ | ■ | • | 2 | 14 | 0.14 |
| 12 | M | 51 | Retromolar | T2N0 | 2 | W | 20 | 10 | D | DD | 48 | Y | 46 | | ▨ | ▨ | ■ | ▨ | | | ▨ | | ▨ | ▨ | ▨ | | ■ | | ■ | | ▨ | ■ | 4 | 10 | 0.4 |
| 13 | M | 77 | Alveolus | T2N0 | 2 | W | 0 | 0 | A | | 60 | Y | 7 | | | ■ | ▨ | | • | • | | | | • | | • | ▨ | • | • | ■ | • | | 2 | 10 | 0.2 |
| 14 | M | 56 | Alveolus | T2N0 | 2 | P | 0 | 0 | D | DD | 18 | Y | 9 | | • | ■ | ▨ | • | • | • | | • | ▨ | • | ▨ | • | ■ | • | • | ■ | ■ | ▨ | 4 | 5 | 0.8 |
| 15 | M | 56 | FOM | T2N0 | 2 | W | 20 | 10 | D | DD | 23 | Y | 12 | ■ | | | | | ▨ | ▨ | ■ | • | • | ▨ | | • | ▨ | | ▨ | | ■ | ▨ | 3 | 10 | 0.3 |
| 16 | M | 64 | Tongue | T2N0 | 2 | W | 40 | 16 | A | | 140 | N | 140 | | | | ▨ | ▨ | | • | ▨ | | ▨ | | ▨ | | | | ■ | ■ | ▨ | | 2 | 12 | 0.17 |
| 17 | M | 58 | Alveolus | T2N0 | 2 | W | 40 | 2 | D | DD | 52 | Y | 20 | | • | ▨ | ▨ | | ▨ | • | ■ | | | ■ | ■ | ■ | ■ | | ▨ | ▨ | | ▨ | 5 | 11 | 0.45 |
| 18 | F | 43 | FOM | T2N0 | 2 | W | 0 | 0 | A | | 48 | N | 48 | | | ▨ | ▨ | | | • | | ■ | | | ▨ | ▨ | | | | ■ | | ▨ | 2 | 13 | 0.15 |
| 19 | F | 72 | Alveolus | T2N0 | 2 | W | 0 | 0 | A | | 42 | N | 42 | | | ▨ | ■ | | | | | ■ | ▨ | ▨ | | ▨ | ■ | | | | | | 3 | 15 | 0.2 |
| 20 | F | 77 | Alveolus | T2N0 | 2 | W | 0 | 0 | D | DD | 24 | Y | 18 | | | | ▨ | ▨ | ■ | ■ | ■ | ■ | ▨ | | | ▨ | | | | ■ | ▨ | | 5 | 14 | 0.38 |
| 21 | M | 34 | Retromolar | T2N1 | 2 | W | 20 | 0 | A | | 110 | Y | 48 | ▨ | | | ▨ | ▨ | | | | ▨ | ▨ | ▨ | | ▨ | | | | | | ▨ | 0 | 11 | 0 |
| 22 | M | 55 | Tongue | T2N1 | 2 | P | 20 | 0 | A | | 48 | Y | 24 | | | | ▨ | ▨ | | ▨ | ▨ | | ▨ | | | | ▨ | | ▨ | | | ■ | 1 | 12 | 0.08 |
| 23 | F | 69 | FOM | T2N0 | 2 | P | 0 | 0 | A | | 52 | N | 52 | | | | | ■ | ▨ | ■ | | | ▨ | | | | | | | ■ | ▨ | | 3 | 16 | 0.18 |
| 24 | M | 73 | FOM | T2N0 | 2 | W | 20 | 0 | A | | 48 | N | 48 | ▨ | • | | ■ | • | ■ | • | ■ | • | • | • | • | ▨ | | ▨ | | • | | | 3 | 8 | 0.37 |
| 25 | F | 65 | FOM | T2N0 | 2 | M | 20 | 12 | D | DD | 3 | Y | 2 | | ■ | ■ | | ▨ | ▨ | ■ | | ▨ | | ▨ | | | ■ | | | ■ | | | 5 | 15 | 0.33 |
| 26 | F | 74 | Buccal | T2N0 | 2 | W | 15 | 0 | D | DD | 12 | N | 12 | ▨ | • | | | | ■ | ▨ | | • | • | | ▨ | ■ | | ▨ | ■ | | | ▨ | 3 | 11 | 0.27 |
| 27 | F | 55 | Buccal | T2N0 | 2 | W | 0 | 0 | A | | 136 | N | 136 | ▨ | • | | ▨ | ■ | • | • | | ▨ | ▨ | ▨ | ■ | ▨ | ▨ | ■ | | | | ▨ | 3 | 9 | 0.33 |
| 28 | F | 35 | Alveolus | T2N1 | 3 | M | 0 | 0 | D | DD | 4 | Y | 1 | ■ | • | ■ | ▨ | ▨ | ▨ | • | ▨ | ▨ | ■ | | | | ▨ | | ▨ | ▨ | ▨ | | 3 | 8 | 0.37 |
| 29 | F | 62 | Alveolus | T3N0 | 3 | W | 0 | 0 | A | | 72 | N | 72 | | | • | | ▨ | | ▨ | ▨ | ▨ | | ▨ | ■ | ■ | ■ | | | | | ▨ | 3 | 12 | 0.25 |
| 30 | M | 73 | Tongue | T2N1 | 3 | W | 60 | 2 | A | | 50 | Y | 36 | ▨ | | ▨ | | | | | ▨ | | | | | | | | | | ■ | | 1 | 16 | 0.06 |
| 31 | M | 31 | Buccal | T2N1 | 3 | W | 0 | 0 | D | DD | 18 | Y | 12 | ▨ | ■ | | | | | | ▨ | | | ▨ | ▨ | ■ | | ■ | ▨ | | ▨ | | 3 | 13 | 0.23 |
| 32 | F | 56 | Tongue | T2N1 | 3 | W | 0 | 0 | D | DD | 36 | Y | 26 | ■ | ■ | | ■ | ■ | ▨ | ▨ | | ▨ | | | ▨ | ▨ | ▨ | | | ▨ | ■ | | 5 | 12 | 0.41 |
| 33 | M | 51 | Buccal | T3N0 | 3 | W | 20 | 2 | D | DD | 1 | Y | 1 | | | | | | ▨ | | ▨ | | ▨ | | | ▨ | | | | | | ▨ | 0 | 14 | 0 |
| 34 | M | 48 | Tongue | T2N1 | 3 | M | 40 | 5 | D | DC | 42 | N | 42 | | | ▨ | ▨ | | ■ | | ■ | | • | ▨ | • | ▨ | | ▨ | ■ | | | ■ | 4 | 12 | 0.33 |
| 35 | F | 36 | Tongue | T2N1 | 3 | M | 30 | 2 | A | | 40 | Y | 6 | ■ | • | ■ | ▨ | ▨ | | | | ▨ | | | ▨ | | | | ▨ | | | | 2 | 13 | 0.15 |
| 36 | M | 76 | Alveolus | T3N0 | 3 | M | 5 | 0 | A | | 166 | N | 13 | | | | | ▨ | | ▨ | | | | ▨ | • | | | | ▨ | ■ | | ▨ | 1 | 13 | 0.07 |
| 37 | M | 50 | Alveolus | T3N0 | 3 | W | 20 | 0 | D | DD | 80 | N | 23 | ▨ | • | | ▨ | ▨ | | ▨ | ▨ | ■ | ■ | ■ | ▨ | | | | ■ | ■ | ■ | | 6 | 12 | 0.5 |
| 38 | M | 55 | Retromolar | T4N2 | 4 | W | 0 | 0 | D | DD | 8 | Y | 4 | ▨ | ■ | | ▨ | ■ | ■ | ■ | ■ | | • | ■ | ▨ | ■ | | ■ | | | ■ | ■ | 10 | 15 | 0.66 |
| 39 | M | 51 | FOM | T4N2 | 4 | W | 20 | 8 | D | DD | 36 | Y | 13 | ▨ | ■ | ■ | ■ | ■ | | ▨ | ▨ | ▨ | ■ | ▨ | ■ | ■ | ▨ | | ■ | ▨ | ▨ | ■ | 9 | 11 | 0.8 |
| 40 | M | 42 | Alveolus | T4N2 | 4 | M | 7 | 2 | D | DD | 12 | Y | 3 | ▨ | ■ | ▨ | | | ▨ | ■ | | ▨ | ▨ | ▨ | ■ | ■ | | | ▨ | ▨ | ■ | | 5 | 11 | 0.45 |
| 41 | M | 84 | FOM | T4N2 | 4 | M | 0 | 4 | D | DD | 4 | Y | 2 | | ■ | ■ | ■ | | ■ | ■ | | | ▨ | ▨ | ▨ | ▨ | ■ | ■ | ■ | ■ | ■ | ■ | 11 | 15 | 0.73 |
| 42 | F | 79 | FOM | T3N0 | 4 | M | 20 | 4 | A | | 36 | Y | 18 | ■ | | | ■ | | | | ▨ | | | | • | | ▨ | ▨ | | | ▨ | ▨ | 2 | 13 | 0.15 |
| 43 | M | 59 | FOM | T4N2 | 4 | W | 30 | 8 | D | DD | 24 | Y | 12 | ▨ | ■ | ■ | ■ | ■ | ■ | | ■ | ▨ | | ■ | ▨ | ▨ | ■ | ■ | ▨ | ■ | | ■ | 11 | 13 | 0.84 |
| 44 | M | 53 | FOM | T3N2 | 4 | W | 20 | 16 | D | DD | 18 | Y | 9 | | ■ | | ▨ | ▨ | | ▨ | | ▨ | | ▨ | | | ■ | ▨ | ■ | | ▨ | ▨ | 3 | 11 | 0.27 |
| 45 | M | 84 | Tongue | T3N1 | 4 | M | 0 | 0 | D | NA | 50 | Y | 6 | ■ | ■ | ▨ | ▨ | | | • | ■ | ▨ | ■ | ■ | ▨ | ▨ | | ▨ | | | | | 5 | 12 | 0.41 |
| 46 | F | 60 | Tongue | T3N2 | 4 | P | 30 | 0 | A | | 28 | Y | 4 | ■ | | | | ▨ | ▨ | | ■ | • | • | ■ | ■ | | ▨ | ▨ | ■ | ▨ | ▨ | ▨ | 2 | 10 | 0.5 |
| 47 | M | 55 | FOM | T4N1 | 4 | W | 10 | 0 | D | NA | 75 | Y | 17 | ■ | | | | | | • | | ▨ | | ▨ | ▨ | ▨ | ■ | | • | ▨ | | | 2 | 12 | 0.16 |
| 48 | M | 56 | Buccal | T4N0 | 4 | M | 0 | 0 | D | DD | 24 | Y | 12 | ■ | | ■ | ■ | | | • | ▨ | ▨ | ▨ | | ▨ | | ■ | • | • | ■ | ■ | ▨ | 6 | 11 | 0.54 |
| **% of informative cases AI** | | | | | | | | | | | | | | 32 | 28 | 34 | 28 | 27 | 29 | 47 | 35 | 25 | 29 | 26 | 31 | 37 | 32 | 38 | 37 | 34 | 36 | 35 | | | |

FOM: floor of mouth; V: verruccous; W: well-differentiated; M: moderately differentiated; P: poorly differentiated; A: alive; D: dead; DD: died of disease; DC: died of other causes; NA: data not available; Y: yes; N: no; FAL: fractional allelic loss. ■ = allelic imbalance; □ = retention of heterozygosity; ▨ = microsatellite instability or non-informative; • = not done.

*© Cancer Research Campaign 1999*

AI. In addition we have determined whether specific patterns of genetic aberration can predict risk of loco-regional recurrence, identify patients with poor outcome and provide additional prognostic information.

### MATERIALS AND METHODS

Forty-eight primary oral SCC were snap frozen in liquid nitrogen immediately after surgical resection and stored at –70°C. Venous blood was stored in tubes with NaCl–EDTA and kept at –20°C until required. Approval for this project was granted by the Ethical Committee at Kings College Hospital. Patients were staged clinically according to Union Internationale Contrele Cancer (UICC) TNM Classification of Malignant Tumours criteria (Hermanek and Sobin, 1987) and restaged following histopathological examination of the resection specimen if the initial nodal status was incorrect. The median follow-up time of the 20 living patients in this study was 49 months (range 28–166 months). The clinicopathological features and risk factors for the tumours studied are summarized in Table 1.

Ten-micrometer frozen sections were mounted onto microscope slides and stained with toluidine blue for microdissection. Samples were digested in 100 μl of lysis buffer (Partridge et al, 1996). Genomic DNA was extracted from venous blood by lysis with Triton-X100. Three polymorphic markers for polymerase chain reaction-restriction fragment length polymorphism (PCR-RFLP) analysis and 13 microsatellite markers, which show frequent loss of heterozygosity when head and neck and oral SCC are examined, were selected for analysis. To examine loss of heterozygosity (LOH) at THRB, D3S686, D3S32 and D3S30 by RFLP analysis of normal and tumour samples was performed using two rounds of PCR analysis (Partridge et al, 1996). Multiple restriction enzymes were used to maximize the number of informative patients for the THRB locus. Amplification was performed in a volume of 50 μl containing 5 μl of DNA solution, or 50 ng of genomic DNA. Fifteen microliters of the product were digested with 10 units of the appropriate restriction enzyme. The digests were fractionated on 4% agarose gels, stained with ethidium bromide and photographed.

PCR primers for the polymorphic microsatellite markers were obtained from Research Genetics, Huntsville, USA or synthesized locally. One of the primers was end-labelled with [$\gamma^{32}$P]ATP and PCR products were generated from standard reactions. Products were separated by gel electrophoresis in denaturing 6% polyacrylamide–7 M urea and autoradiographed overnight. Labelled pBR322 was included as a sequencing ladder to facilitate sizing of the alleles.

Cases were scored by visual inspection of band patterns and considered to show AI if the ratio of the two alleles in the tumour was 50% less than that detected for the normal sample. Novel microsatellite alleles were identified by the presence of bands that were absent in the normal sample, and these cases scored as showing microsatellite instability and excluded from the informative cases. The FAL score was calculated for each patient by dividing the number of loci showing AI by the number of informative loci.

The relationship between two ordinal variables, or two quantitative variables, was investigated using Spearman's rank correlation. Ordinal or quantitative variables were compared between different categories defined by a nominal variable by the Kruskal–Wallis test (for three or more groups) and the Mann–Whitney *U*-test (for two groups). If three or more groups were compared, the Mann-Whitney *U*-test was only applied if the Kruskal–Wallis test was significant. The $\chi^2$ test was used to compare proportions, except in the case of a 2 × 2 table with any expected value less than 5; in this case Fisher's exact test was employed. Yates' correlation was applied when analysing 2 × 2 tables using the $\chi^2$ test. To investigate the relationship between risk factors (tobacco intake and alcohol consumption) and FAL score, patients were divided into groups. These were: non-smokers, 1–25 cigarettes a day and > 25 cigarettes a day; no alcohol, 1–4 units a day, 5–9 units a day and > 10 units a day. Survival curves were calculated using the Kaplan–Meier product-limit method and compared by the log-rank test. Hazard ratios, an 'average' relative event rate over the follow-up period, were calculated using Cox's regression (Cox, 1972). For example, if every month there was one death in a group and two deaths in another, then the hazard ratio for the second group relative to the first would be 2.

### RESULTS

### Relationship between AI and clinicopathological features of the tumours examined

The highest frequency of AI was detected for markers at D3S1562 (47%), the interferon alpha (INF-α) locus at 9p21 (37%), D9S43 at 9p13 (38%), D9S177 at 9q22 (37%), p53 (36%) and DCC (34%). Overall 45/48 (94%) of the lesions examined showed AI at one or more of the chromosomal loci studied. The data are summarized; Table 1 and Figure 1 show representative examples of the microsatellites used. No relation was observed between the

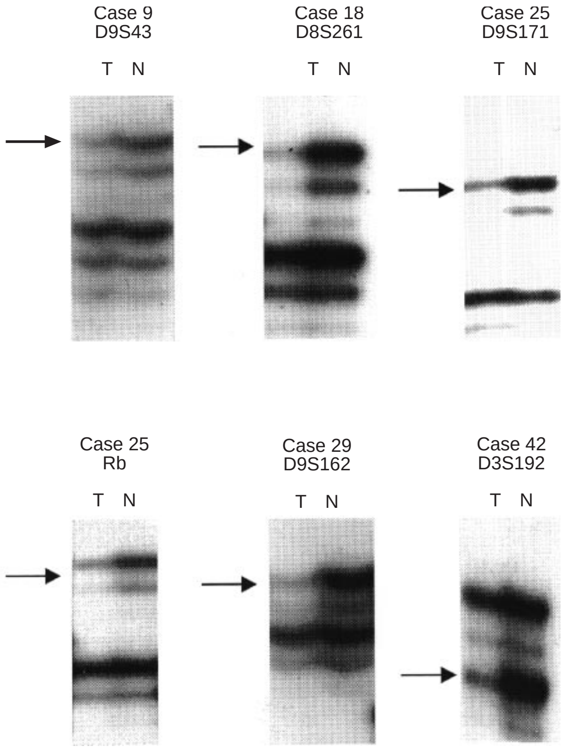

**Figure 1** Microsatellite alterations in DNA from tumour (T). The loci and case numbers are indicated above the gel lanes. Normal leucocyte DNA is indicated by N. The arrows highlight the altered alleles

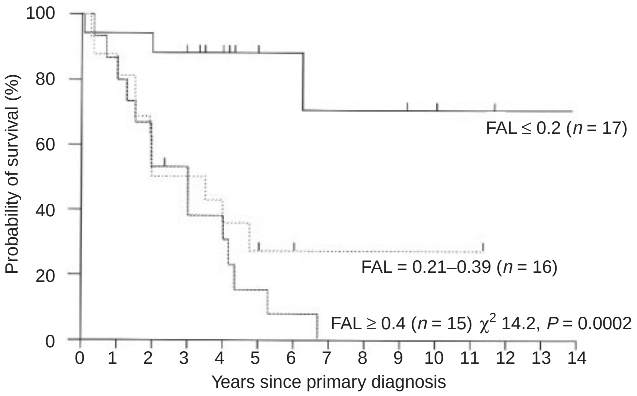

**Figure 2** Kaplan–Meier analysis of patients with oral squamous cell tumours. Patients were divided into three groups according to the FAL score and compared. Bars on the survival curves indicated patients alive at the indicated time

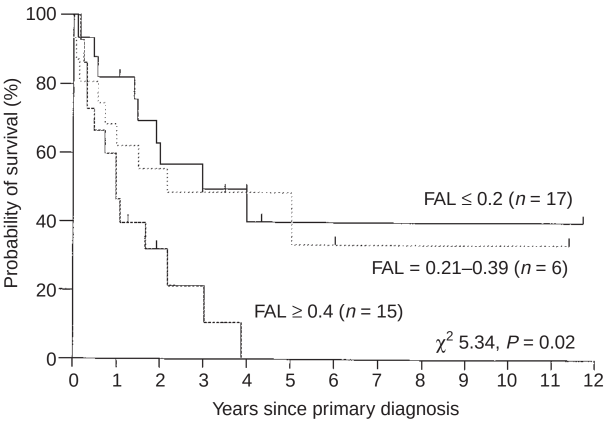

**Figure 3** Probability of developing local recurrence for the three FAL score groups median FAL score and the site of the tumour. Lesions examined developed on the floor of the mouth (13), alveolus (12), on the buccal mucosa (9), tongue (11) and at other sites (4). The median FAL at these four sites was 0.37, 0.30, 0.27, 0.18 and 0.20 respectively. Similarly, no relationship was found between exposure to risk factors (smoking and alcohol consumption), higher tumour grade and the median FAL.

### Relationship between FAL score and outcome

The median FAL was 0.29 (range 0–0.84). However, the FAL score for patients presenting with stage 4 tumours was much higher, median 0.50 (range 0.15–0.84), than the score for patients presenting with stage 1–3 tumours, median 0.27 (range 0–0.80, $P = 0.01$). To investigate the relationship between the frequency of genetic aberrations and overall survival, patients were divided into three approximately equally sized groups on the basis of the FAL score. Figure 2 shows the survival probability for the three groups and reveals a significant association between the FAL score and overall survival. The relative hazard ratios for survival analysis in the low, intermediate and high FAL score groups were 1, 5.37 and 8.36, respectively ($P < 0.0002$), using the group with the lowest level of AI as the reference group. Multivariate analysis using Cox's regression, indicates that this relationship is independent of stage. (The $P$-value was 0.0006 when allowing for stage.) A significant relationship was also detected between the FAL score and development of loco-regional recurrence (see Figure 3). Univariate analysis was performed to assess whether AI at sequences within the p53, Rb and DCC tumour suppressor genes, or at one or more loci within the candidate suppressor areas, were associated with poor prognosis. (The results for several adjacent loci at the same chromosomal region, which together form these candidate tumour suppressor gene areas, were combined to reduce multiple significance testing, see Table 2.) However, as ten or more patients were non-informative for all but the THRB locus, tests of the prognostic significance of AI are inevitably weak. Nevertheless, AI at one or more loci within the 3p24–26 region, 3p21, 3p13, 9p21 and within the THRB and DCC genes were all associated with reduced survival ($P < 0.1$, Table 2).

AI within the three chromosomal regions investigated at 3p that were associated with poor prognosis ($P < 0.1$ on univariate analysis), were examined to determine whether they were of inde-

**Table 2** Relationship between outcome and AI at the chromosomal loci tested

| Chromosomal region | Loci examined | Number of cases non-informative | HR for survival | *P*-value |
|---|---|---|---|---|
| 3p24–26 | D3S192, THRB | 4 | 4.21 | 0.0002 |
| 3p21 | D3S32, D3S1241, D3S1562 | 0 | 1.99 | 0.07 |
| 3p13 | D3S1296. D3S1562, D3S30 | 2 | 2.52 | 0.02 |
| 8p12–23 | D8S261, D8S298, Ank-1 | 5 | 1.25 | ns |
| 9p21 | D9S162, INFα, D9S171 | 1 | 2.65 | 0.01 |
| 9p13 | D9S43 | 9 | 1.56 | ns |
| 9q22.3 | D9S177 | 14 | 1.78 | ns |
| 13q14.2 | Rb | 7 | 1.16 | ns |
| 17p13.1 | p53 | 12 | 1.85 | ns |
| 18q21.1 | DCC | 13 | 2.39 | 0.05 |

HR = hazard ratio.

© Cancer Research Campaign 1999

pendent prognostic significance. AI at 3p24–26, 3p13 and 9p21 were found to predict poor prognosis independently, of each other with hazard ratios of 3.93, 2.60 and 2.48 ($P = 0.002$, $P = 0.03$ and $P = 0.03$ respectively). This implies that patients with AI at all of these regions at 3p would have an approximately 25 times increase in their mortality rate relative to a patient showing retention of heterozygosity at the loci tested. AI at all of these three key chromosomal regions was also found to be a better predictor of outcome than TNM stage, which no longer reached statistical significance when the effect of aberrations at these three regions was taken into account. AI at these three chromosomal regions was also found to predict prognosis independent of the FAL score; however, FAL was still significant once these loci had been allowed for, suggesting that aberrations affecting other chromosomal regions may still be important.

### Commonly occurring patterns of AI

The data were analysed using the $\chi^2$ test to determine whether AI at pairs of loci occurred more frequently than might be expected by chance. There were 171 possible pairs of loci for which associations could be tested. Approximately nine significant pairings would be expected to be present by chance at the 5% level and 14 were actually found. Two of these were significant at $P = 0.001$, AI at D3S686 and D9S171 and D9S162 and INF-α. A further three associations were significant at $P = 0.01$ and formed a triplet comprising D3S1296, D9S43 and DCC.

Further analysis was performed to see whether AI at loci at different chromosomal arms, which frequently occur together, was associated with poor survival. Twenty-six cases were assessable at both D3S686 and D9S171, and 21 cases at D3S1296, DCC and D9S43. When cases with AI at D3S686 and D9S171 were compared with those that showed retention of heterozygosity (ROH) using Cox's regression, AI involving this doublet was found to be associated with a poorer prognosis ($P = 0.02$) and to be a better predictor of outcome than the FAL score or TNM stage. In addition, AI at D3S1296 and DCC, D3S1296 and D9S43, and DCC and D9S43 were all found to be associated with poor prognosis ($P < 0.001$) and to predict prognosis more accurately than the FAL score or TNM stage.

### DISCUSSION

AI at all loci tested was detected for patients with early stage 1 and 2 tumours and for advanced stage 3 and 4 lesions. A variety of different patterns of AI were seen for tumours of the same TNM stage. Although these aberrations represent only a fraction of the total number of abnormalities likely to be present in each cancer, the different profiles for each tumour reveal the potential complexity of the molecular process involved in head and neck tumorigenesis and provide a partial explanation for the different biological behaviour that tumours classified as being of the same TNM stage may exhibit.

The percentage AI at the different chromosomal segments demonstrates large variation amongst the published series for head and neck (El-Naggar et al, 1993; Ah-See et al, 1994; Nawroz et al, 1994; Field et al, 1996; Partridge et al, 1996). The percentage AI found for the present study of oral SCC are typically lower than those reported for head and neck SCC. However, since the various studies have applied different markers, with different levels of informativeness, this precludes the conclusion that these differences reflect distinct mechanisms of tumour development.

Summarizing the number of loci showing AI as a FAL score has identified a subgroup of patients likely to develop locoregional recurrence ($P = 0.02$, Figure 2) and show reduced survival ($P = 0.0002$, Figure 3), thus providing new prognostic information. These findings extend the report by Field et al, (1996), indicating that FAL greater than median value correlated with poor survival ($P < 0.19$). However, these authors also found a significant relationship between FAL score and the presence of lymph nodes at presentation, an association not confirmed in the present study. These differences are likely to reflect inclusion of cases previously treated by radiotherapy in the study reported by Field et al (1966). The FAL score was not related to tobacco usage and alcohol consumption. Lack of correlation between these risk factors and AI at chromosome 3p and 9p has been reported previously for oral SCC (Partridge et al, 1994) and pre-invasive lesions (Mao et al, 1996). This suggests that other aetiological agents, environmental factors and inherited factors, which have yet to be determined, often exert their effects via the same genetic mechanisms.

Univariate analysis revealed that AI at 3p24–26, 3p21, 3p13 and 9p21 and DCC predict poor prognosis. However, AI at 3p24–26, 3p13 and 9p21 were found to predict prognosis independently of each other with hazard ratios of 3.93, 2.60 and 2.48. This indicates that patients with AI at each of these regions have an approximately 25 times increase in their mortality rate relative to a patient showing ROH at these loci. AI at these three key chromosomal regions was also found to be a better predictor of outcome than TNM stage, which no longer reached statistical significance when the effect of aberrations at these three regions was taken into account.

AI at specific pairs of loci, namely D3S686 and D9S171, D3S1296 and D9S43, D3S1296 and DCC, and D9S43 and DCC, also provided additional prognostic information. AI at these doublets occurred more frequently than would be expected by chance and was associated with reduced survival. For example, AI involving the doublets D3S686 and D9S171, and D9S162 and INF-α (adjacent loci at 9p21), and at least two of D3S1296, DCC and D9S43, was found to be a better predictor of prognosis than the FAL score or TNM stage. This suggests that these chromosomal regions must contain, or be close to, sequences that, when both are disrupted, have a very adverse effect on outcome for this group of patients.

At present, the identity of the sequences at these key chromosomal loci is largely unknown. The p16 gene, a cyclin-dependent kinase inhibitor, is a well-defined tumour suppressor gene at 9p21. This sequence lies centromeric to D9S162 and INF-$\alpha$, two markers found to be significantly associated with poor prognosis in the present study. However, the pattern of AI found at 9p when a series of overlapping markers is used to study upper aerodigestive tract tumours, suggests the existence of additional tumour suppressor genes centromeric (Kim et al, 1997; Weist et al, 1997) and telomeric (Neville et al, 1995; Kim et al, 1997) to p16. The high frequency of AI at the D9S43 marker, which lies centromeric to p16, in the present study strengthens the notion that this chromosomal region harbours additional tumour suppressor sequences for this tumour type.

AI at 3p21.33, the location of the D3S686 marker, has been extensively studied and several sequences have been isolated from deletions involving this region (reviewed by Todd et al, 1997). The

© Cancer Research Campaign 1999

widely expressed semaphorin genes H Sema IV and Sema V have been identified as the targets for deletion in lung cancer cell lines showing homozygous deletions at 3p21.33 (Sekido et al, 1996). These sequences are widely expressed in adult tissues, which suggests that they may serve functions other than nerve cone guidance, perhaps influencing contact inhibition or differentiation in epithelia. However, many other sequences have been identified as possible targets for deletion within this gene-rich area (Todd et al, 1996) and the role of these sequences in head and neck SCC is currently unknown. The 3p13 region, at which the D3S1296 marker resides, contains the U2020 small cell lung cancer deletion and lies telomeric to the 3p14.3 fragile site. This region is also likely to harbour a tumour suppressor gene for oral cancer.

Several breakpoints involving 18q have also been reported for head and neck SCC suggesting the possible involvement of more than one gene at this chromosomal arm. The DCC, DPC4 and MADR2 genes (Frank et al, 1997) have all been implicated as putative tumour suppressor genes at 18q21. Although AI involving DCC occurs frequently in solid tumours this observation does not establish DCC as a tumour suppressor gene and the low frequency of AI at DPC4 (Kim et al, 1996) suggests that LOH at this region may be associated with other tumour suppressor genes.

Typically, tumour suppressor genes act in a recessive manner such that loss of both alleles is required to inactivate these sequences. However, the findings from this study reveal that loss of one copy of a tumour suppressor gene or a candidate suppressor area provides additional prognostic information. At present the status of the other allele of the p16, p53, DCC and Rb tumour suppressor sequences investigated in this study is unknown. However, several reports have shown that AI within a known tumour suppressor sequence can occur without mutation of the other copy (Wagata et al, 1993; Lee et al, 1994; Li et al, 1995; Maestro et al, 1996). There are also other ways of inactivating tumour suppressor genes; for example, silenced gene transcription associated with hypermethylation (Jones et al, 1990), or association with oncogenic viral proteins (Min et al, 1994). Nevertheless, the finding that information about aberrations affecting just one copy of a tumour suppressor gene sequences does have prognostic significance, suggests that so-called dosage effects, where levels of the gene product are altered sufficiently to disrupt normal growth control processes (Kemp et al, 1993), have significant biological consequences and may be more important than previously realized.

These data reveal that summarizing the level of genetic damage as a FAL score, as well as screening tumours for AI at key chromosomal loci, and pairs of loci can provide new prognostic information. This suggests that, as more of the critical aberrations associated with tumorigenesis are identified, it will be possible to develop a molecular staging system, which will identify patients with an unfavourable genetic and clinical profile who are likely to benefit from aggressive treatment when they first present at the clinic. However, many studies have evaluated single, or small groups of markers for head and neck cancer patients, and as a result of this clinicians are now faced with a daunting list of publications describing new prognostic markers. At this stage careful consideration must be given to how, and if, this information, which is often obtained following analysis of very small numbers of patients, can be used to influence treatment decisions. What is clear is that, as our knowledge of the complexity of the events in tumorigenesis has increased, multiple markers will be needed to give representative information about any given tumour. At this stage large numbers of cases need to be examined and new prognostic markers validated by testing a different geographic population before this information can be used to influence decisions about treatment.

### ACKNOWLEDGEMENT

This research was supported by a S Thames R&D Project Grant.

### REFERENCES

Ah-See KW, Cooke TG, Pickford IR, Soutar D and Balmain A (1994) An allelotype of squamous carcinoma of the head and neck using microsatellite markers. *Cancer Res* **54**: 1617–1621

Anneroth G, Batsakis J and Luna M (1987) Review of the literature and a recommended system of malignancy grading in oral squamous cell carcinomas. *Scand J Dental Res* **95**: 229–249

Brennan JA, Boyle JO, Koch W, Goodman SN, Hruban RH, Eby YJ, Couch MJ, Forastiere AA and Sidransky D (1995) Association between cigarette smoking and mutation of the p53 gene in squamous cell carcinoma of the head and neck. *N Engl J Med* **332**: 712–717

Califarno J, van der Reit P, Westra W, Nawroz H, Clayman G, Piantadosi S, Corio R, Lee D, Greenberg B, Koch W and Sidransky D (1996) Genetic progression model for head and neck cancer: implications for field cancerisation. *Cancer Res* **56**: 2488–2492

Cairns P, Polascik TJ, Eby Y, Tokino K, Califarno J, Merlo A, Mao L, Herath J, Jenkins R, Westra W, Rutter JL, Bucker A, Gabrielson E, Tockman M, Cho KR, Hedrick L, Bova GS, Isaacs W, Koch W, Schwab D and Sidransky D (1995) Frequency of homozygous deletion at p16/CDKN2 in primary human tumours. *Nature Genet* **11**: 210–212

Cox DR (1972) Regression models and life tables (with discussion). *J Roy Stat Soc B* **34**: 187–220

El-Naggar AK, Lee MS, Wang G, Luna MA, Goepfert H and Batsakis JG (1993) Polymerase chain reaction-based restriction length polymorphism analysis of the short arm of chromosome 3 in primary head and neck squamous cell carcinoma. *Cancer* **72**: 881–886

El-Naggar AK, Hurr K, Batsakis JG, Luna MA, Goepfert H and Huff V (1995) Sequential loss of heterozygosity at microsatellite motifs in preinvasive head and neck squamous carcinoma. *Cancer Res* **55**: 2656–2659

Field JK, Tsiiriytos C and Zoumpourlis V (1994) Allele loss on chromosome 3 in squamous cell carcinoma of the head and neck correlates with poor clinical prognostic indicators. *Int J Oncol* **4**: 543–549

Field JK, Kiaris H, Tsiriyotis C, Adamson R, Zoumpourlis V, Rowley H, Taylor K, Whittaker J, Howard P, Beirne JC, Gosney JR, Woolgar J, Vaughan ED, Spandidos DA and Jones AS (1995) Allelotype of squamous cell carcinoma of the head and neck: fractional allelic loss correlated with survival. *Br J Cancer* **72**: 1180–1188

Frank CJ, McClatchey KD, Davaney KO and Carey TE (1997) Evidence that loss of chromosome 18q is associated with tumour progression. *Cancer Res* **57**: 824–827

Hermanek P and Sobin L (1987) *UICC TNM Classification of Malignant Tumours*, 3rd edn. Spinger-Verlag: Berlin

Ishwad CS, Ferrell RE, Rossie KM, Appel BN, Johnson JT and Myers EN (1996) Loss of heterozygosity of the short arm of chromosomes 3 and 9 in oral cancer. *Int J Cancer* **69**: 1–4

Jones PA and Buckley TD (1990) The role of DNA methylation in cancer. *Adv Cancer Res* **54**: 1–23

Kelker W, van Dyke DL, Worsham M, Christopherson PL, James CD, Conlon MR and Carey TE (1996) Loss of 18 and homozygosity for the DCC locus: possible markers for clinically aggressive squamous cell carcinoma. *Anticancer Res* **16**: 2363–2371

Kemp CJ, Donehower LA, Bradley A and Balmain A (1993) Reduction of p53 gene dosage does not increase initiation or promotion but enhances malignant progression of chemically induced skin tumours. *Cell* **74**: 813–822

Kerangueven F, Toguchida T, Coulier F, Allione F, Wargniez V, Simony-Lafontaine J, Longy M, Jacquemier J, Sobol H, Eisinger F and Birnbaum D (1997) Genome-wide search for loss of heterozygosity shows extensive genetic diversity of human breast carcinomas. *Cancer Res* **57**: 5469–5474

Kiaris H, Jones AS, Spandidos DA, Vaughan ED and Field JK (1994) Loss of heterozygosity on chromosome 8 in squamous cell carcinomas of the head and neck. *Int J Oncol* **5**: 1243–1248

 *© Cancer Research Campaign 1999*

Kim SK, Fan Y, Papadimitrakopoulou V, Clayman G, Hittelman WN, Hong WK Lotan R and Mao L (1996) DPC4, a candidate tumour suppressor gene, is altered infrequently in head and neck squamous cell carcinoma. *Cancer Res* **56**: 2519–2521

Kim SK, Ro JY, Kemp BL, Lee SJ, Kwoon TJ, Fong KM, Sekido Y, Minna JD, Hong WK and Mao L (1997) Identification of three distinct tumour suppressor gene loci on the short arm of chromosome 9 in small cell lung cancer. *Cancer Res* **52**: 400–403

Langdon JD and Henk JM (1995) Classification and staging. In *Malignant Tumours of the Mouth, Jaws and Salivary Glands*, Langdon JD and Henk JM (eds), pp. 36–49. Edward Arnold: London

Largey JS, Meltzer SJ, Yin J, Norris K, Sauk JJ and Archibald DW (1993) Loss of heterozygosity of p53 in oral cancers demonstrated by the polymerase chain reaction. *Cancer* **71**: 1933–1937

Lee DL, Koch WM, Yoo G, Lango M, Reed A, Califarno J, Brennan JA, Westra WH, Zahurak M and Sidransky D (1997) Impact of chromosome 14q loss on survival in primary head and neck squamous cell carcinoma. *Clin Cancer Res* **3**: 501–505

Lee NK, Ye YW, Li X, Schweitzer C and Nisen PD (1994) Allelic loss on chromosome 13 can proceed histological changes in head and neck cancer. *Int J Oncol* **5**: 205–210

Li Y-Q, Pavelic ZP, Wang L-J, McDonald JS, Gleich L, Munck-Wikland E, Dacic S, Danilovic Z, Pavelic LJ, Wilson KM, Gluckman JL and Stambrook PJ (1995) Altered p53 in microdissected, metachronous, premalignant and malignant oral lesions from the same patients. *J Clin Pathol (Clin Mol Pathol)*, **48**: M269–M272

Lydiatt WM, Davidson BJ, Schantz SP, Caruana S and Chaganti RSK (1998) 9p21 deletion correlates with recurrence in head and neck cancer. *Head Neck* **20**: 113–118

Maestro R, Piccinin S, Doglioni C, Gasparotto D, Vukosavljevic G, Sulfaro S, Barzan L and Boiocchi M (1996) Chromosome 13q deletion mapping in head and neck squamous cell carcinomas: identification of two distinct regions of preferential loss. *Cancer Res* **56**: 1146–1150

Mao L, Fan JS, Ro JY, Batsakis JG, Lippman S, Hittelman W and Hong WK (1996) Frequent microsatellite alterations at chromosomes 9p21 and 3p14 in oral premalignant lesions and their value in cancer risk assessment. *Nature Med* **2**: 682–685

Min BM, Baek JH, Shin KH, Gujuluva CN, Cherrick HM and Park NH (1994) Inactivation of the p53 gene by either mutation of HPV infection is extremely frequent in human oral squamous cell carcinoma cell lines. *Eur J Cancer B Oral Oncology* **5**: 338–435

Nawroz H, van der Reit P, Hruban RH, Koch W, Rupert JM and Sidransky D (1994) Allelotype of head and neck squamous cal carcinoma. *Cancer Res* **54**: 1152–1155

Neville EM, Stewart M, Myskow M, Donnelly RJ and Field JK (1995) Loss of heterozygosity at 9p23 defines a novel locus in non small cell lung cancer. *Oncogene* **11**: 581–585

Nylander K, Schildt EB, Eriksson M, Magnusson A, Mehle C and Roos G (1996) A non-random deletion in the p53 gene in oral squamous cell carcinoma. *Br J Cancer* **73**: 1381–1386

Partridge M, Kiguwa S and Langdon JD (1994) Frequent deletion of chromosome 3p in oral squamous cell carcinoma. *Eur J Cancer B Oral Oncol* **30B**: 248–251

Partridge M, Emilion G and Langdon JD (1996) LOH at 3p correlates with a poor survival in oral squamous cell carcinoma. *Br J Cancer* **73**: 366–371

Rowley H, Jones AS and Field JK (1995) Chromosome 18: a possible site for a tumour suppressor gene deletion in squamous cell carcinoma of the head and neck. *Clin Otolaryngol* **20**: 266–277

Sekido Y, Lader S, Latif F, Chen J-Y, Duh F-M, Wei M-H, Albanesi JP, Lee C-C, Lerman MI and Minna JD (1996) Human semaphorins A(v) and IV reside in the 3p21.3 small cell lung cancer deletion region and demonstrate distinct expression patterns. *Proc Natl Acad Sci USA* **93**: 4120–4125

Spiro RH, Huvos AG, Wong GY, Spiro JD, Gnecco CA and Strong EW (1986) Predictive thickness in squamous carcinoma confined to the tongue and floor of mouth. *Am J Surg* **152**: 345–350

Tobe T and Ishizaki K (1993) Loss of 17p, mutation of the p53 gene, and overexpression of the p53 protein in oesophageal squamous cell carcinoma. *Cancer Res* **53**: 846–850

Todd S, Franklin WA, Varella-Garcia M, Kennedy T, Hiliker CE, Hahner L, Anderson M, Weist JS, Drabkin HA and Gemmil RM (1997) Homozygous deletions of human chromosome 3p in lung tumours. *Cancer Res* **57**: 1344–1352

van der Reit P, Nawroz H, Hruban RH, Corio R, Tokino K, Koch W and Sidransky D (1994) Frequent loss of chromosome 9p21–22 in head and neck cancer progression. *Cancer Res* **54**: 1156–1158

van Dyke DL, Worsham MJ, Benninger MS, Krause CJ, Baker SR, Wolf GT, Drumheller T, Tilley BC and Carey TE (1994) Recurrent cytogenetic abnormalities in squamous cell carcinomas of the head and neck region. *Genes Chromosom Cancer* : **9**: 192–206

Vogelstein B, Fearnon ER, Kern S, Hamilton SR, Preisinger AC, Nakamura Y and White R (1989) Allelotype of colorectal carcinomas. *Science* **244**, 207–211

Wagata T, Shibagaki I, Imamura M, Shimada Y, Toguchida J, Yandell DW, Ikenaga M, Wistuba II, Montellano FD and Milchgrub S (1993) Deletions of chromosome 3p are frequent early events in the pathogenesis of uterine cervical carcinoma. *Cancer Res* **57**: 3154–3158

Weist JS, Franklin JT, Otstot JT, Forbey K, Varella-Gracia M, Rao K, Drabkin H, Gemmil R, Ahrendt S, Sidransky D, Saccomanno G, Fountain JW and Anderson WW (1997) Identification of a novel region of homozygous deletion on chromosome 9p in squamous cell carcinoma of the lung: the location of a putative tumour suppressor gene. *Cancer Res* **57**: 1–6

Wu CL, Sloan P, Read AP, Harris RH and Thakkar NS (1994) Deletion mapping on the short arm of chromosome 3 in oral squamous cell carcinoma of the oral cavity. *Cancer Res* **54**: 6484–6488

*© Cancer Research Campaign 1999*
